# Applicability of the Meniscus-Removal Method for Q-Band Liquid Characterization in Semi-Open Waveguide Cell †

**DOI:** 10.3390/s23125390

**Published:** 2023-06-07

**Authors:** Michał Kalisiak, Wojciech Wiatr, Radosław Papis

**Affiliations:** Institute of Electronic Systems, The Faculty of Electronics and Information Technology, Warsaw University of Technology, 00-665 Warsaw, Poland; w.wiatr@elka.pw.edu.pl (W.W.); rad.papis@gmail.com (R.P.)

**Keywords:** complex permittivity, microwave measurements, vector network analyzer, scattering parameters, WR-22, phase ambiguity

## Abstract

We present the broadband transmission-reflection meniscus-removal method for liquid characterization in a semi-open rectangular waveguide. The algorithm utilizes 2-port scattering parameters measured with a calibrated vector network analyzer for three states of the measurement cell: empty and filled with two liquid levels. The method enables the mathematical de-embedding of a symmetrical sample of a liquid, not distorted with a meniscus, and provision of its permittivity and permeability, as well as its height. We validate the method for propan-2-ol (IPA), a 50% aqueous solution of IPA, and distilled water in the Q-band (33–50 GHz). We investigate typical problems for in-waveguide measurements, such as phase ambiguity.

## 1. Introduction

Accurate knowledge of complex liquid permittivity is needed in many fields of science and technology [1], such as medicine [2], biology, chemistry, agriculture, radio communications, remote sensing, etc. In the microwave band, the precise permittivity determination is usually performed with a vector network analyzer (VNA) connected to a dedicated test fixture designed for a specific band and measurement technique, such as resonant, transmission/reflection (T/R) and reflection coefficient (1-port) methods [1].

T/R methods, based on two-port scattering matrix measurement, enable comprehensive characterization of the permittivity and permeability of a liquid tested over a broad frequency range. There are two main types of fixtures employed in T/R measurements: double plug [3,4,5,6,7], and semi-open [8,9,10]. In the first, the volume of liquid is set and determined by the fixed dielectric plugs. In the second, equipped with just one plug, the liquid partly fills the cell, and its volume can be easily adjusted, allowing one to perform multistate measurements (of different volumes).

**Figure 1:**
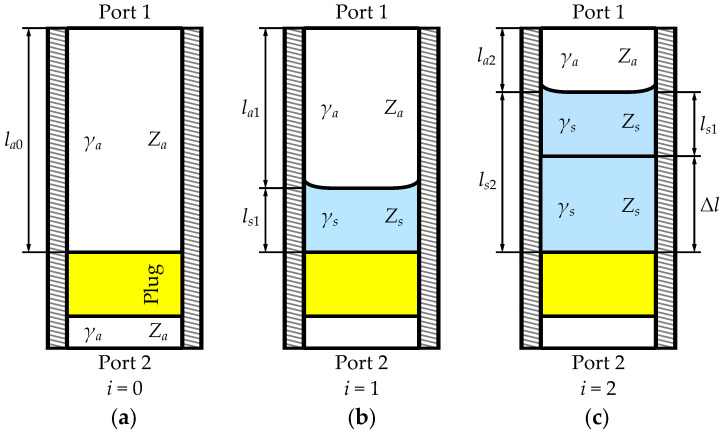
Sketch of the semi-open rectangular waveguide fixture in its three measurement states of liquid under test distorted by the meniscus: (**a**) the empty cell; (**b**) the initial volume of a liquid; (**c**) the final volume. List of symbols: $l_{ai}$—the length of *i*-th airline section; $l_{si}$—the height of *i*-th sample; Δl—the height increment; $Z_{a}$, $Z_{s}$—wave impedances; and $\gamma_{a}$, $\gamma_{s}$—propagation constants for the air and sample, respectively.

However, the surface of a liquid in a semi-open cell is not ideally flat but distorted with a meniscus, as illustrated in Figure 1. Therefore, the measured sample generally becomes asymmetrical. In our previous paper [10], we proposed a new method for dealing with the asymmetry of the distorted sample. It employs three measurement states, an empty cell and a cell with two different volumes of a liquid (Figure 1), for which we assume the reproducible shape of the meniscus. Using S matrices measured for all three states, we de-embed the additional portion of the poured liquid, which is ideally symmetrical. The de-embedded T-matrix of the increment volume of the liquid enables calculation of the height increment and the sample permittivity and permeability. This method was introduced for coaxial test cells and tested in the 0.1–18 GHz bandwidth.

This paper extends the meniscus-removal method for higher frequencies, the 33–50 GHz (Q-band). This is not straightforward, as, apart from requiring more complicated formulas, in-waveguide material characterization creates challenges that do not usually occur in coaxial cells operating from low frequencies, such as phase ambiguity [4,8,11,12,13,14,15,16,17,18,19,20,21,22,23,24,25]. We used propan-2-ol (IPA), distilled water and a 50% aqueous solution of propan-2-ol (IPA50). For the Q-band measurements, we manufactured a novel semi-open fixture in a WR-22 rectangular waveguide standard, the preliminary design of which was presented in [26].

The structure of the paper is as follows: In Section 2, we introduce the meniscus-removal method for in-waveguide permittivity and permeability measurements. The concept and design of the rectangular waveguide fixture used for the measurements are presented in Section 3. The results of liquid microwave characterization are shown in Section 4. We conclude the work in Section 5.

## 2. Theory

In this section, mathematical algorithms used in the data processing are presented. We start with general formulas regarding rectangular waveguides, which leads to the presentation of the meniscus-removal method for a waveguide application. Although the method has already been presented in [10] for coaxial cells, in this paper, we present the whole algorithm for the reader’s convenience in Section 2.1 with all the differences in waveguide approach, especially the phase ambiguity problem (Section 2.2), considered.

Propagation constant of the waveguide γ=α+jβ, where α is the attenuation and β is a phase constant, is defined as [16,27,28]:

(1) $$\gamma^{2}=k_{c}^{2}-k^{2}\;,$$

(2) $$k_{c}=\frac{2\pi }{\lambda_{c}}\;,$$

(3) $$k=\frac{\omega }{c}\sqrt{\mu_{r}\varepsilon_{r}}\;,$$

(4) $$\lambda_{c}=\frac{2}{\sqrt{{\left(\frac{g}{a}\right)}^{2}+{\left(\frac{h}{b}\right)}^{2}}}\;,$$

where $\varepsilon_{r}=\varepsilon_{r}^{\prime }-j\varepsilon_{r}^{\prime \prime }$—relative complex permittivity with *j*—imaginary unit; $\mu_{r}=\mu_{r}^{\prime }-j\mu_{r}^{\prime \prime }$—relative complex permeability; $k_{c}$—cutoff wavenumber; *k*—wavenumber; ω=2πf—angular frequency; *c*—speed of light in a vacuum; $\lambda_{c}$—cutoff wavelength; *a*, *b*—lengths of the wider and narrower walls of the waveguide, respectively. In general, g,h∈N, for fundamental mode in a rectangular waveguide—$TE_{10}$ (transverse electric)—g=1 and h=0; thus, $\lambda_{c}=2a$.

In-waveguide sample measurements are typically described with the transfer matrices (T), which are related to the scattering (S) matrix as follows:

(5) $$T=\left[\begin{array}{cc} T_{11} & T_{12}\\ T_{21} & T_{22} \end{array}\right]=\frac{1}{S_{21}}\left[\begin{array}{cc} -\det S & S_{11}\\ -S_{22} & 1 \end{array}\right]\;,\;S=\frac{1}{T_{22}}\left[\begin{array}{cc} T_{12} & \det T\\ 1 & -T_{21} \end{array}\right]\;.$$

In transfer matrix notation, the transmission through the uniform transmission line of length *l* and propagation constant γ is described by a diagonal matrix

(6) $$T_{tline}=\left[\begin{array}{cc} e^{-\gamma l} & 0\\ 0 & e^{\gamma l} \end{array}\right]\;.$$

The transition (with zero electrical length) from medium *a* to medium *s* defines the matrix

(7) $$T_{sa}=\frac{1}{\sqrt{1-\Gamma_{sa}^{2}}}\left[\begin{array}{cc} 1 & \Gamma_{sa}\\ \Gamma_{sa} & 1 \end{array}\right]\;.$$

The reflection coefficient of the flat and transversal boundary between media *a* and *s* is given by

(8) $$\Gamma_{sa}=\frac{Z_{s}-Z_{a}}{Z_{s}+Z_{a}}=-\Gamma_{as}\;,$$

where *Z* is the wave impedance defined by the ratio of the transverse components of the electric and magnetic fields, and, for TE waves, equals [16,22,28]

(9) $$Z=\frac{E_{x}}{H_{y}}=-\frac{E_{y}}{H_{x}}=\frac{j\omega \mu }{\gamma }\;.$$

### 2.1. Meniscus-Removal Method

The meniscus-removal method utilizes scattering matrices of the fixture in three states: the empty cell and with two levels of liquid, as pictured in Figure 1. Measurements are performed with a calibrated VNA, with reference planes at the connectors of the fixture. The *k*-th state, transformed to transfer matrix notation (5), can be modeled as follows

(10) $$T_{fi}=T_{ci}T_{b}=T_{ai}T_{\langle si\rangle }T_{b}\;,$$

where $T_{ci}$ models the interesting part of the fixture, the cell itself—the part above the plug. $T_{ai}$ describes the transmission through the airline section by (6) with the length $l_{ai}$ and the known propagation constant of air with relative permittivity $\varepsilon_{ra}$

(11) $$\gamma_{a}=\sqrt{k_{c}^{2}-\varepsilon_{ra}{\left(\frac{\omega }{c}\right)}^{2}}\;;$$

$T_{b}$ models transmission through the bottom part of the fixture including the transition from the air to the plug, transmission through the plug, transition from the plug to the air, and transmission through the bottom airline section towards port 2. $T_{\langle si\rangle }$ describes the transmission through the sample-filled line of the propagation constant $\gamma_{s}$ and length $l_{si}$ referred to the air; thus, surrounded with transitions from the air to the sample, $T_{sa}$, and vice-versa, $T_{as}$,

(12) $$T_{\langle si\rangle }={\tilde{T}}_{sa}T_{si}T_{as}\;.$$

The tilde over ${\tilde{T}}_{sa}$ represents the meniscus distortion.

In the case of the empty cell, $T_{\langle s0\rangle }=I$, where I is the 2×2 identity matrix. Thus, (10) reduces to:

(13) $$T_{f0}=T_{a0}T_{b}\;,$$

where the length of the empty cell $l_{a0}$ is known from the design. Expression (13) enables de-embedding from (10), the interesting part, the cell itself, $T_{ci}$, for i=1,2. Thus, $T_{ci}$ can be calculated from

(14) $$T_{ci}=T_{fi}T_{f0}^{-1}T_{a0}\;,$$

and modeled as

(15) $$T_{c1}=T_{a1}T_{\langle s1\rangle }=T_{a2}T_{a\Delta }T_{\langle s1\rangle }\;,$$

(16) $$T_{c2}=T_{a2}T_{\langle s2\rangle }=T_{a2}T_{\langle s1\rangle }T_{\langle s\Delta \rangle }\;.$$

In (15) and (16), $T_{a\Delta }$ models the transmission through the airline section and the $T_{\langle s\Delta \rangle }$ transmission through the sample with reference to the wave impedance of the air $Z_{a}$,

(17) $$T_{\langle s\Delta \rangle }=T_{sa}T_{s\Delta }T_{as}=T_{sa}T_{s\Delta }T_{sa}^{-1}\;.$$

$T_{a\Delta }$ and $T_{s\Delta }$ are expressed with (6), where the length is

(18) $$\Delta l=l_{s2}-l_{s1}=l_{a1}-l_{a2}\;,$$

since the height increment of the liquid sample results in the same length decrement of the airline section. Therefore, $T_{\langle s1\rangle }$ contains the factor distorted by the meniscus, while $T_{\langle s\Delta \rangle }$ models a symmetrical line filled with the liquid sample. Compiling (15) and (16), we get

(19) $$T_{\langle s\Delta \rangle }=T_{c1}^{-1}T_{a\Delta }T_{c2}\;.$$

After multiplication in (19), we obtain

(20) $$T_{\langle s\Delta \rangle }=\left[\begin{array}{cc} T_{22c}^{\left(1\right)}T_{11c}^{\left(2\right)}e^{-\gamma_{a}\Delta l}-T_{12c}^{\left(1\right)}T_{21c}^{\left(2\right)}e^{\gamma_{a}\Delta l}\; & T_{22c}^{\left(1\right)}T_{12c}^{\left(2\right)}e^{-\gamma_{a}\Delta l}-T_{12c}^{\left(1\right)}T_{22c}^{\left(2\right)}e^{\gamma_{a}\Delta l}\\ T_{11c}^{\left(1\right)}T_{21c}^{\left(2\right)}e^{\gamma_{a}\Delta l}-T_{21c}^{\left(1\right)}T_{11c}^{\left(2\right)}e^{-\gamma_{a}\Delta l}\; & T_{11c}^{\left(1\right)}T_{22c}^{\left(2\right)}e^{\gamma_{a}\Delta l}-T_{21c}^{\left(1\right)}T_{12c}^{\left(2\right)}e^{-\gamma_{a}\Delta l}\; \end{array}\right]\;,$$

where $T_{pqc}^{\left(i\right)}$ are the adequate parameters of $T_{ci}$ for p,q=1,2. In (20), Δl is the only unknown parameter. From the symmetry condition, $T_{12}=-T_{21}$, in T-matrix notation, we get

(21) $$\exp\left(2\gamma_{a}\Delta l\right)=\frac{T_{22c}^{\left(1\right)}T_{12c}^{\left(2\right)}-T_{21c}^{\left(1\right)}T_{11c}^{\left(2\right)}}{T_{12c}^{\left(1\right)}T_{22c}^{\left(2\right)}-T_{11c}^{\left(1\right)}T_{21c}^{\left(2\right)}}=r\;,$$

and by calculating the median value (which is more robust to high errors and excesses than the mean value) of

(22) $$\Delta l\left(f\right)=\frac{\ln r}{2\gamma_{a}}\;$$

we determine the optimal increment height of the sample Δl.

Then, after determining the trace of (17) and (19)

(23) $$\mathrm{tr}\;T_{\langle s\Delta \rangle }=\exp\left(-\gamma_{s}\Delta l\right)+\exp\left(\gamma_{s}\Delta l\right)=2\cosh\gamma_{s}\Delta l\;,$$

we calculate the propagation constant of the liquid sample

(24) $$\gamma_{s}=\frac{1}{\Delta l}\mathrm{arcosh}\left(\frac{1}{2}\mathrm{tr}\;T_{\langle s\Delta \rangle }\right)\;,$$

that is not disturbed by the meniscus.

For a nonmagnetic sample, $\mu_{rs}=1$, the permittivity can be determined using (1)–(3):

(25) $$\varepsilon_{rs}=\left(k_{c}^{2}-\gamma_{s}^{2}\right)\frac{c^{2}}{\omega^{2}}\;.$$

For a magnetic sample, we should also calculate the reflection coefficient for a transition from air to the sample $\Gamma_{sa}$. Inserting (9) to (8) for the boundary air-sample leads to

(26) $$\Gamma_{sa}=\frac{\mu_{rs}\frac{\gamma_{a}}{\gamma_{s}}-1}{\mu_{rs}\frac{\gamma_{a}}{\gamma_{s}}+1}\;,$$

and solving for $\mu_{r}$

(27) $$\mu_{rs}=\frac{1+\Gamma_{sa}}{1-\Gamma_{sa}}\frac{\gamma_{s}}{\gamma_{a}}\;.$$

The permittivity is then calculated again from (1)–(3)

(28) $$\varepsilon_{rs}=\frac{k_{c}^{2}-\gamma_{s}}{\mu_{rs}}\frac{c^{2}}{\omega^{2}}\;.$$

To obtain the reflection coefficient $\Gamma_{sa}$, we translate (17) to

(29) $$T_{t}T_{s\Delta }=T_{\langle s\Delta \rangle }T_{t}\;.$$

Using additional markings $T_{pqs}$ for the adequate parameters of $T_{\langle s\Delta \rangle }$ for p,q=1,2, that correspond to the measured values in (19), we get

(30) $$\left[\begin{array}{cc} e^{-\gamma_{s}\Delta l}\; & \Gamma_{sa}e^{\gamma_{s}\Delta l}\\ \Gamma_{sa}e^{-\gamma_{s}\Delta l}\; & e^{\gamma_{s}\Delta l} \end{array}\right]=\left[\begin{array}{cc} T_{11s}+\Gamma_{sa}T_{12s}\; & T_{12s}+\Gamma_{sa}T_{11s}\\ T_{21s}+\Gamma_{sa}T_{22s}\; & T_{22s}+\Gamma_{sa}T_{21s} \end{array}\right]\;.$$

Finally, by equating the terms $T_{21}$ of the matrices in (30), we extract the reflection coefficient at the ideal air-sample boundary

(31) $$\Gamma_{sa}=\frac{T_{21s}}{e^{-\gamma_{s}\Delta l}-T_{22s}}\;,$$

to calculate $\mu_{rs}$ and $\varepsilon_{rs}$ from (27) and (28).

### 2.2. Phase Ambiguity

In waveguide material characterization, there are several challenges arising from phase ambiguity [4,8,11,12,13,14,15,16,17,18,19,20,21,22,23,24,25]. Let us consider a typical case for the NRW method [11,22,29], with propagation factor *P* defined as

(32) $$P=\left|P\right|\exp\left(j\varphi_{P}\right)=\exp\left(-\gamma_{s}l_{s}\right)\;.$$

The phase of the propagation factor $\varphi_{P}$ should be negative, continuous in frequency, and decreasing with a longer sample. However, without any further processing, the measured phase is in the range $\left(-\pi ,\;\pi \right]$. Thus, when extracting $\gamma_{s}$, the phase ambiguity problem arises on the imaginary part of the propagation constant (phase constant)

(33) $$\gamma_{s}=-\frac{\log\left(P\right)}{l_{s}}=-\frac{1}{l_{s}}\left[\ln\left|P\right|+j\left(\varphi_{P}+2\pi n\right)\right]\;.$$

Functions $\log\left(\cdot \right)$ and $\ln\left(\cdot \right)$ represent the complex and real natural logarithms. In general, *n* can be an integer, but, for “normal” wave propagation, the imaginary part of $\gamma_{s}$ should be positive; thus, n≤0. In our meniscus-removal method, $\gamma_{s}$ is calculated from the $\mathrm{arcosh}\left(\cdot \right)$ function, but the analysis remains analogous.

The two main problems highlighted in the literature [4,8,11,12,13,14,15,16,17,18,19,20,21,22,23,24,25] are:

1. continuous phase while counting the logarithm;
2. the proper *n* for the lowest frequency of measurement $f_{min}$.

The first point also relates to measurements in coaxial cells and is rather easy to achieve by unwrapping the phase (detecting a jump in phase greater than π between two subsequent frequencies and shifting the phase by 2π in the opposite direction) [16,25], and assuring the frequency step is low enough not to introduce phase changes higher than π in the transmission coefficient. Other methods for tracking group delay are proposed in [11,12,16,17,22].

The second point can be troublesome to fulfill, especially for high-permittivity liquids in high-frequency bands. It can be avoided by choosing a maximum sample length $l_{s\;max}$ for measurements starting at frequency $f_{min}$ [22]

(34) $$l_{s\;max}=\frac{\lambda_{gs}\left(f_{min}\right)}{2}\;,$$

where

(35) λgs=2πImγs

is the transmission line guide wavelength in the measured sample [15].

In [11], the ambiguity of the low-frequency value while solving $\gamma_{s}$ is not mentioned, but the samples measured by Weir are shorter than $l_{s\;max}$ (34). In [8], when calculating the length with knowledge of the air’s propagation constant, Somlo used an initial approximation of air length. Ogunlande et al. in [4] used a sample permittivity prediction method to choose the proper solution of the logarithm. Varadan and Ro in [18] proposed using the Kramers–Kronig relation [17,30].

This work proposes two algorithms to determine the initial phase properly. In the case of measurements of length related to propagation in air, the situation is quite simple, because, for an improper logarithm solution, the calculated airline section length (22) depends on the frequency, so it is enough to find *n* for which the expression $\max\left[\Delta l(f)\right]-\min\left[\Delta l(f)\right]$ is minimal. For a proper (24) solution, we use an initial guess of $\varepsilon_{rs}$ and $\mu_{rs}$ at a starting frequency known from a literature model or extrapolated from measurements in lower bands; then, we predict the number of waves inside the sample.

## 3. Design of the Rectangular Waveguide Fixture

**Figure 2:**
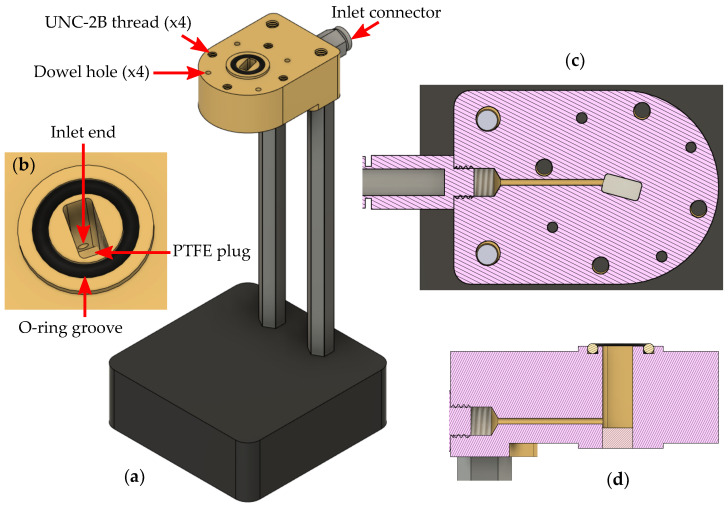
(**a**) Model of the semi-open rectangular waveguide test cell for liquid permittivity measurements on a stand, (**b**) its interior close-up, (**c**) horizontal and (**d**) vertical cross-section.

The fixture design is based on a section of the rectangular waveguide for the Q-band, WR22 [31], presented in Figure 2. Its structure is adapted to the circular flange type UG-383/U with four UNC-2B threads and four holes for centering dowels; thus, it remains compatible with standard waveguides, adapters, etc. The internal cross-section of the waveguide has dimensions of 5.690×2.845 mm. For vacuum, the operating frequency range extends from 33 to 50 GHz; the cutoff frequencies for the lowest order and the next mode are 26.346 GHz, 52.692 GHz, respectively.

The liquid can be poured through the inlet, visible in Figure 2c, located at the bottom of the cell, which has an inner diameter of 1 mm. The diameter should be small enough not to disturb the EM field and big enough for convenient liquid dosing. The influence on the permittivity measurement of the inlet was investigated in [26]. The volume of liquid can be changed or adjusted without any disassembling. Since all the cables and connectors remain intact, the measurement results are expected to exhibit higher consistency. With dosing liquid, its column height gradually increases. We assume its surface shape to be repeatable. Excess air flows out through a small hole in a narrower wall of an additional waveguide section connected above the fixture.

**Figure 3:**
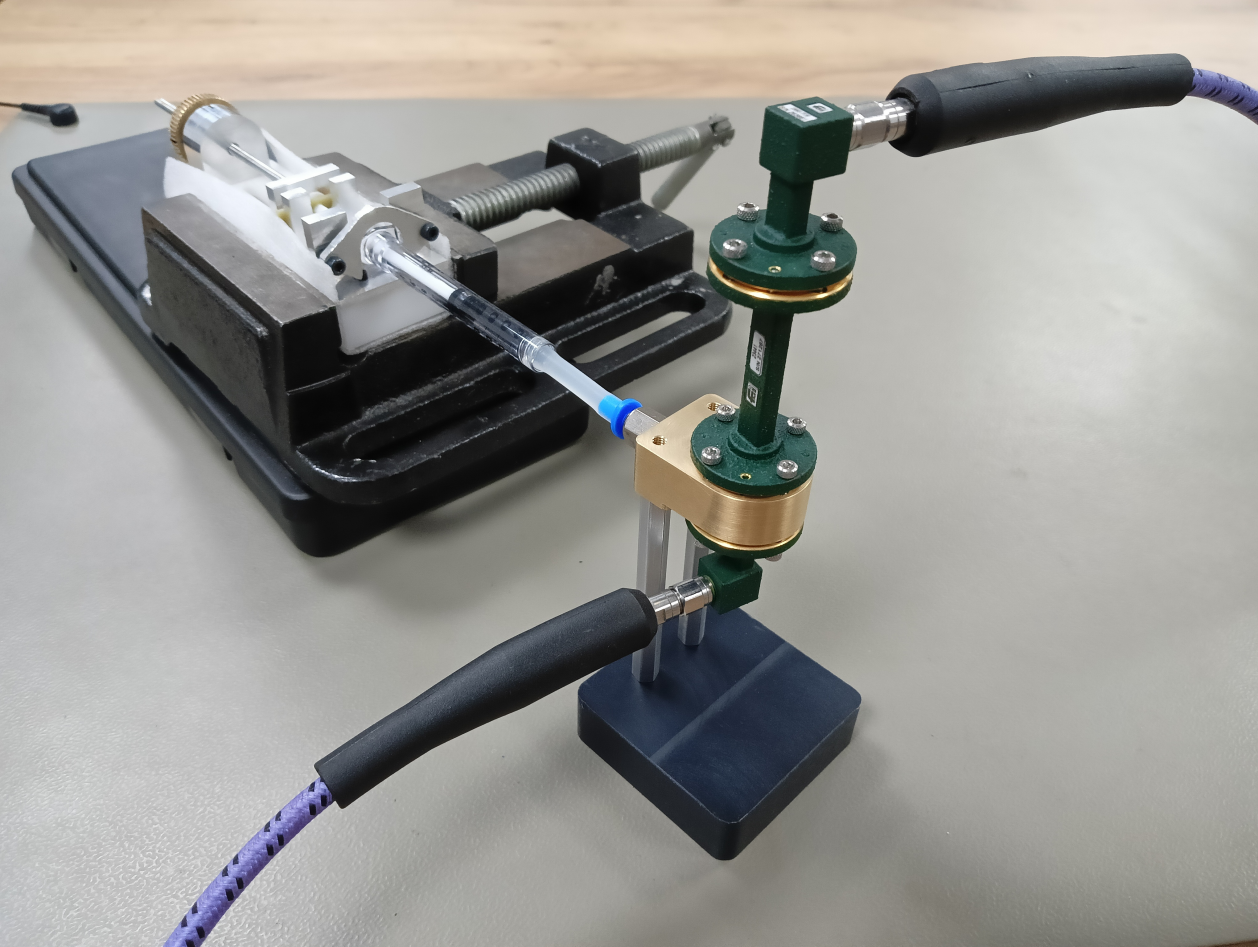
Measurement setup: the semi-open rectangular waveguide fixture connected to the VNA, with the liquid dosing appliance.

The manufactured fixture used in the measurement system is presented in Figure 3. The fixture was milled from brass (alloy CuZn40Pb2) with a computer numeric control (CNC) milling machine. The precision of milling was about 0.05 mm. The most important part of the fixture—the waveguide interior—was precisely cut out using Wire EDM (electrical discharge machining) with a precision of about 0.02 mm.

The total length of the fixture was 15.00 mm, limited by the milling process. The dielectric plug from polytetrafluoroethylene (PTFE) holding the liquid sample was 2.96 mm long and was flush with the fixture’s bottom edge. Therefore, the length of the cell $l_{a0}$ was 12.04 mm. To have an opportunity to measure larger volumes of liquid with this fixture, the additional waveguide section can extend the cell. To seal it, a groove for an o-ring rubber gasket was provided (Figure 2b). Therefore, depending on the needs, the port 1 calibration plane can be at the top or bottom of the additional waveguide. The waveguide is integrated with a stand assuring vertical orientation crucial for reducing the effects of the higher modes, which was analyzed in our previous work [32].

## 4. Experimental Results

Two-port measurements were performed with a VNA Rohde & Schwarz ZVA50 in the 33–50 GHz frequency range. A picture of the measurement system is shown in Figure 3. The signal was transmitted via coaxial cables in 2.4 mm standard and then converted to a WR-22 system with the adapters. The VNA was calibrated in the frequency range from 33 to 50 GHz with the through-reflect-line (TRL) method [33], using a line with a physical length of 2.97 mm ± 0.05 μm. The through was realized as a direct connection of the bottom adapter and the bottom plane of the additional waveguide piece (flush through), while a flush short served as a reflect.

To ensure that the measurement errors are small, the calibration should be performed meticulously, minimizing any movement of the cables, and keeping the connectors as close to the place of subsequent measurements as possible. The dosing of the liquid should be conducted slowly to ensure the most repeatable shape of the liquid sample surface, and to not introduce undesirable air bubbles. The initial level and the increment of the liquid column height should be appropriately adapted to the properties of the liquid, such as its attenuation.

We measured the scattering matrices of the fixture in three states, the empty cell and with two levels of liquid, to apply the meniscus-removal method, as described in Section 2. We examined three liquids: IPA, distilled water, and a 50% aqueous solution of IPA (IPA50). The solution was prepared with the definition of the volume fraction [34], the volume of IPA and the volume of distilled water prior to mixing were equal. The temperature in the laboratory was 24 °C.

**Figure 4:**
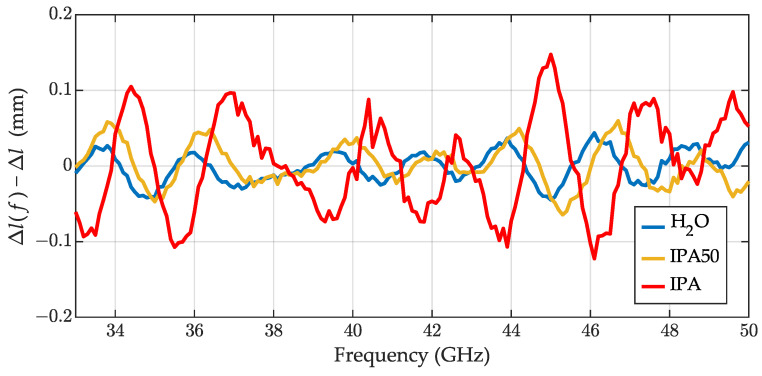
The errors in the sample height determination defined as $\Delta l\left(f\right)-\Delta l$ for water (blue line), 50% aqueous solution of IPA (yellow line), and IPA (red line).

In the first step, we determined the height of the sample increment at each frequency $\Delta l\left(f\right)$ (22) and the optimal value Δl. The calculated lengths were 2.56 mm, 1.49 mm, and 1.19 mm for IPA, IPA50 and water, respectively. Figure 4 presents the errors $\Delta l\left(f\right)-\Delta l$, which probably resulted from residual VNA calibration errors.

**Figure 5:**
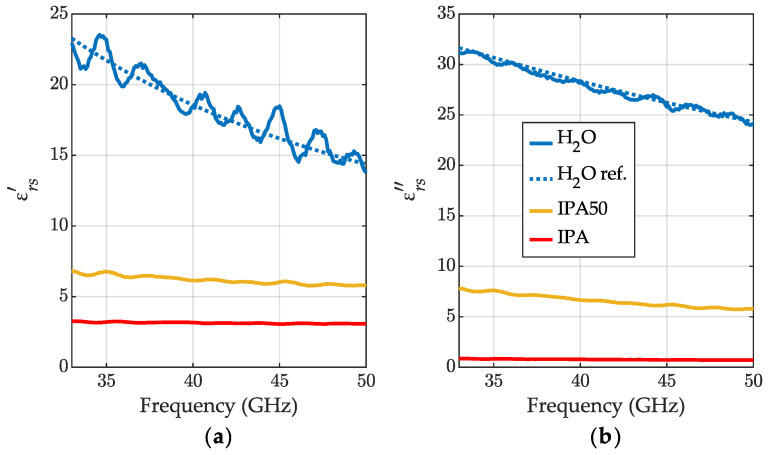
The relative permittivity at 24 °C: (**a**) the real part $\varepsilon_{rs}^{\prime }$ and (**b**) the imaginary part $\varepsilon_{rs}^{\prime \prime }$ obtained for the meniscus-removal method for distilled water (blue lines), for 50% (the volume fraction) aqueous solution of IPA (IPA50) (yellow lines) and for IPA (red lines). The reference data for water permittivity [35] (blue dotted lines).

In Figure 5, we present the relative permittivity of the liquids obtained with (25), assuming their relative permeability $\mu_{rs}=1$. The permittivity values calculated for IPA and IPA50 are presented with red and yellow lines, respectively. Unfortunately, we did not find any reference data in this frequency range to compare the results against—we can only say that the characteristics were relatively smooth. The permittivity acquired for water revealed significant ripples correlated with the length errors from Figure 4. The ripples probably resulted from residual VNA calibration errors. As reference data for water, we used [35].

**Figure 6:**
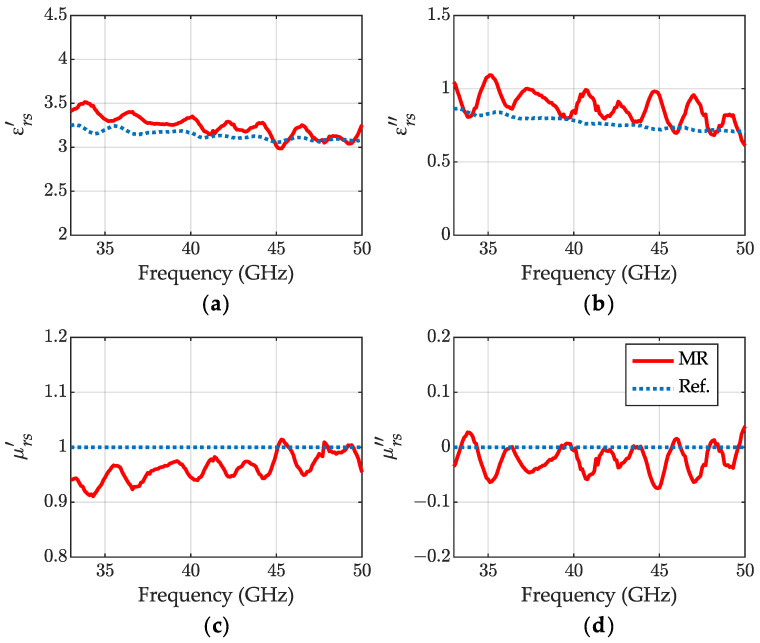
The relative permittivity (**a**) $\varepsilon_{rs}^{\prime }$, (**b**) $\varepsilon_{rs}^{\prime \prime }$ and permeability (**c**) $\mu_{rs}^{\prime }$, (**d**) $\mu_{rs}^{\prime \prime }$ of IPA at 24 °C obtained for the meniscus-removal method (MR) (red lines). Blue dotted lines—the reference: $\varepsilon_{rs}$—the non-magnetic results from Figure 5 and $\mu_{rs}=1$.

The results without any assumption regarding the permeability value are presented in Figure 6 for IPA. The calculations rely more on the reflection measurements (27) and (28), and expose more ripples than non-magnetic results, that are correlated with the ripples from length determination (Figure 4).

## 5. Discussion and Conclusions

In this work, we presented the broadband transmission-reflection meniscus-removal algorithm for liquid characterization, previously applied for coaxial test cells [10], here extended for in-waveguide measurements in the 33–50 GHz bandwidth (Q-band). Utilizing scattering parameters measured with calibrated VNA for three states of the cell, empty and with two levels of liquid, we were able to mathematically de-embed the symmetrical sample of liquid, not distorted with a meniscus, and provide the sample’s permittivity and permeability, as well as its height. We performed the measurements using a newly designed and manufactured rectangular waveguide test cell. The cell allows multistate 2-port measurements with a calibrated VNA and is compatible with the standard WR-22 waveguide flange. We investigated the typical problem for in-waveguide measurements—phase ambiguity—and provided the solution.

We performed measurements for propan-2-ol, a 50% aqueous solution of IPA, and distilled water. The measurements obtained for water with the high real part of permittivity $\varepsilon_{rs}^{\prime }$ and high losses $\varepsilon_{rs}^{\prime \prime }$ revealed the limitations of the method and relatively high errors due to residual VNA calibration errors. Generally, the higher losses and the lower volumes of liquid can be measured to provide reasonably high values of transmission $\left|S_{21}\right|$. Further research could contain an error analysis of the method for both the sample length determination (including mechanical measurements for comparison) and the permittivity.

## Data Availability

The data presented in this paper are available on request from the corresponding author.
